# Novel Human Rhinoviruses and Exacerbation of Asthma in Children^1^

**DOI:** 10.3201/eid1411.080386

**Published:** 2008-11

**Authors:** Nino Khetsuriani, Xiaoyan Lu, W. Gerald Teague, Neely Kazerouni, Larry J. Anderson, Dean D. Erdman

**Affiliations:** Centers for Disease Control and Prevention, Atlanta, Georgia, USA (N. Khetsuriani, X. Lu, N. Kazerouni, L.J. Anderson, D.D. Erdman); Emory University School of Medicine, Atlanta (W.G. Teague); 2 Current affilation: California Department of Public Health, Richmond, California, USA.

**Keywords:** human rhinoviruses, human rhinovirus genogroup C, human rhinovirus species A, human rhinovirus species B, bronchial asthma, rhinovirus PCR, rhinovirus VP1 sequences, dispatch

## Abstract

To determine links between human rhinoviruses (HRV) and asthma, we used data from a case–control study, March 2003–February 2004, among children with asthma. Molecular characterization identified several likely new HRVs and showed that association with asthma exacerbations was largely driven by HRV-A and a phylogenetically distinct clade of 8 strains, genogroup C.

Human rhinovirus (HRV) infection triggers asthma exacerbation (*1*), but there are no data on links between specific HRVs and asthma. Molecular sequence–based methods enabled recent identification of several novel HRVs (*2*–*9*) and have made it practical to look for genogroup and genotype-specific correlations with disease. In a previous study, we found a significantly higher prevalence of HRVs in children with asthma exacerbations than in children with well-controlled asthma (*10*). In this study, we used molecular characterization methods to examine HRVs from these patients with asthma.

## The Study

The case–control study was conducted in metropolitan Atlanta, Georgia, USA, during March 2003–February 2004, among children with asthma who were >2 years of age (*10*). Case-patients were defined as patients with asthma exacerbation; controls were defined as patients with stable asthma. Information on symptoms of acute viral respiratory illness was also collected. The definitions, epidemiologic and laboratory methods, and clinical description of patients are available from Table 1 and the previously published report (*10*).

**Table 1:** Criteria and definitions used in the study of children with asthma, March 2003–February 2004 (*10*)

Category	Criteria
Current persistent asthma:	
In children 2–5 y of age	All of the following: 1. Physician diagnosis of asthma 2. >2 previous episodes of cough, wheeze, and/or respiratory distress 3. Current treatment with asthma medications 4. Parent or sibling with current or past diagnosis of asthma or allergy, and/or current or past evidence of atopy (defined by seasonal rhinitis, eczema, or food hypersensitivity)
In children 6–17 y of age	All of the following: 1. Physician diagnosis of asthma 2. Symptoms of asthma in the past 12 mo 3. Current treatment with asthma medications
Case (asthma exacerbation)	Current persistent asthma, hospital admission or clinic visit for asthma exacerbation, and all of the following: 1. Signs and symptoms of airflow obstruction (i.e., cough, wheeze, shortness of breath, chest tightness) within past 48 h 2. Increased asthma symptoms resulting in hospital admission or clinic visit 3. Repeated use of short-acting β-agonists within past 48 h 4. Increased dose or addition of a new asthma controller therapy within past wk
Control (well-controlled asthma)	Current persistent asthma, routine clinic visit for asthma, and all of the following: 1. No systemic steroid therapy in past 4 wk 2. No increase in dose and no new controller medications in past wk 3. No change in the frequency of use of short-acting rescue medications in past wk 4. No increase in asthma symptom frequency in past wk
Acute respiratory viral illness	>2 of the following: fever, stuffy/runny nose, headache, muscle aches, and pain or redness of eye(s) at the time of clinic visit or hospital admission

HRVs were detected in nasopharyngeal swab specimens by seminested reverse transcription–PCR (RT-PCR) targeting the 5′-noncoding region (NCR) (*10*). For further genetic characterization, HRV-positive samples were extracted from a previously unopened aliquot and amplified by using a nested RT-PCR that targeted the virus capsid protein 1 (VP1) gene at positions 2432–2781, based on HRV 1B (GenBank accession no. D00239) for species A and positions 2531–2799, based on HRV 14 (GenBank accession no. NC_001490) for species B. We used Sequencher 3.1.1 software (Gene Codes, Ann Arbor, MI, USA) for sequence assembly and editing. Nucleotide and predicted amino acid sequences were aligned with previously published HRV VP1 sequences (GenBank accession nos. AY355180–AY3552831, EF186077, EF077279, EF077280, EF582385–EF582387) by using ClustalW as implemented in BioEdit (version 7.0.5) (www.mbio.ncsu.edu/BioEdit/bioedit.html).

Phylogenetic trees were constructed by using the neighbor-joining algorithm implemented in PAUP* version 4.0.d10 (*11*). Partial VP1 sequences for the novel HRV strains were submitted to GenBank (accession nos. EU312093–EU312101).

As reported previously (*10*), HRVs were detected by a 5′-noncoding region seminested RT-PCR in 53 (37%) of 142 children with asthma, including 39 (60%) of 65 case-patients and 14 (18%) of 77 controls. Of these, the HRVs from 29 (55%) (24 [62%] of the 39 HRV-positive case-patients and 5 [36%] of the 14 HRV-positive controls) were subsequently genotyped. VP1 sequences from the remaining 24 HRV-positive specimens could not be obtained because of low amplicon yield (Table 2). Specimens from patients with symptoms of acute viral respiratory infection (Table 1) were more likely than those from patients without viral symptoms to yield sufficient VP1 amplicon for genotyping (percent genotyped 85% and 36%, respectively; odds ratio [OR] 9.1; 95% confidence interval [CI] 2.1–50.0; p<0.05).

**Table 2:** Human rhinoviruses identified in 53 pediatric patients with asthma, March 2003–February 2004, Atlanta, Georgia, USA*

HRVs	Receptor-binding group	No. among all HRV+ patients, n = 53	No. among HRV+ case-patients, n = 39		No. among HRV+ controls, n = 14
			Virus symptoms, n = 20	No virus symptoms, n = 19	
Total no. genotyped†		29	17	7	5
Species A		18	12	3	3
HRV12	Major	1	1	0	0
HRV30	Minor	2	2	0	0
HRV36	Major	1	0	1	0
HRV39	Major	1	0	0	1
HRV43	Major	1	1	0	0
HRV44	Minor	2	1	0	1
HRV46	Major	1	1	0	0
HRV49	Minor	2	1	1	0
HRV53	Major	1	0	1	0
HRV54	Major	1	1	0	0
HRV61	Major	1	1	0	0
HRV65	Major	1	1	0	0
HRV66	Major	1	0	0	1
HRV68	Major	1	1	0	0
GA23584‡	Unknown	1	1	0	0
Species B		3	1	0	2
HRV48	Major	1	0	0	1
HRV99	Major	2	1	0	1
Genogroup C§	Unknown	8	4	4	0
Not genotyped	Unknown	24	3	12	9

*HRV, human rhinovirus; case-patients, asthma patients with exacerbations; controls, asthma patients without exacerbation. †HRV genotype based on partial virus capsid protein (VP1) gene sequence. Serotype designation based on >90% VP1 amino acid sequence identity with respective prototype strains. ‡Strain GA23584 showed 73.0% amino acid sequence identity with HRV80. §Genogroup C HRVs form a clade phylogenetically distict from species A and B HRVs.

Of the 29 HRVs successfully genotyped, species A accounted for 18 (62%) strains, species B accounted for 3 (10%), whereas 8 (28%) strains formed a phylogenetically distinct clade, which we provisionally named “genogroup C” (Table 2, Figure). Of the 18 HRV-A strains, 17 showed close genetic relatedness (80.7%–93.8% nucleotide and 89.6%–98.8% predicted amino acid sequence identity) to HRV prototype strains. One HRV-A strain (GA23584) was highly divergent from the closest prototype, HRV80 (73.2% nucleotide and 73.0% amino acid sequence identity), which suggests that it could represent a distinct previously undescribed HRV. The 3 HRV-B strains were closely related to prototype strains (84.0%–88.6% nucleotide and 89.7%–93.4% predicted amino acid sequence identity).

**Figure:**
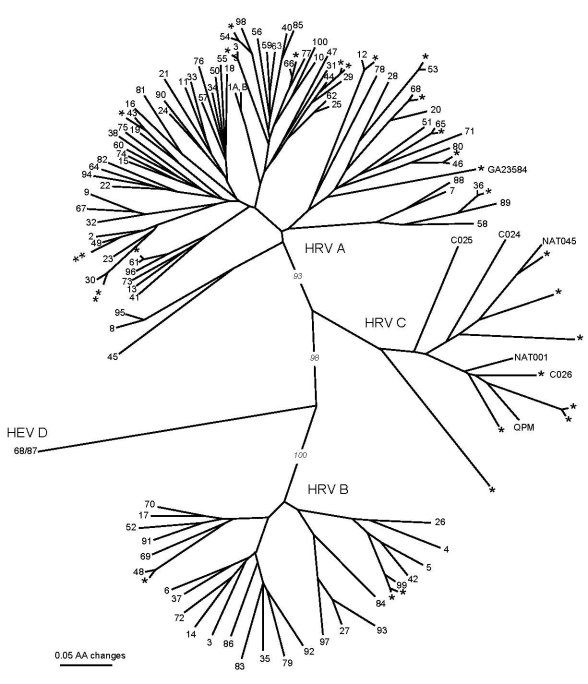
Phylogenetic tree of partial virus capsid protein 1 (VP1) amino acid sequences of human rhinoviruses (HRVs) identified in 29 HRV-positive pediatric asthma patients, March 2003–February 2004, Atlanta, Georgia, USA (designated *), previously published sequences of strains QPM (GenBank accession no. EF186077), C024-C026 (accession nos. EF582385–EF582387), and NAT001 and NAT045 (accession nos. EF077279–EF077280). HRV prototype strains designated 1A, 1B, 2-100. Human enterovirus (HEV) 68/HRV87 (designated 68/87) is included as outgroup. Tree construction and bootstrap values determined with PAUP* (*11*).

The partial VP1 sequences of genogroup C strains were phylogenetically distinct from HRV species A and B and showed a substantial intragroup diversity (Figure). VP1 sequence identity of these viruses with the closest match within the same genogroup ranged from 68.4% to 74.6% for nucleotide and from 68.5% to 85.5% for amino acid sequences. These novel viruses were related to other recently described HRVs: HRV–QPM detected in specimens from Australia (*4*), C024–C026 detected in specimens from Hong Kong (*6*), and NAT001 and NAT045 detected in specimens from California (*8*) (Figure). Their identity scores compared with HRV–QPM were 66.0%–82.7% for nucleotide and 65.2%–86.9% for amino acid sequences. One of the strains (GA23592) was almost identical in partial VP1 sequence to C026 (Figure). The degree of genetic diversity among the genogroup C viruses far exceeded that between HRVs defined as distinct serotypes by classical serologic methods, which suggests that at least 7 of 8 of these viruses are antigenically distinct from each other rather than minor variants of the same serotype. The genogroup C HRV identity scores were substantially lower when compared with their closest matches from species A and B: 48.2%–51.1% for nucleotide and 38.5%–49.8% for amino acid sequences, and 35.9%–42.8% for nucleotide and 29.3%–35.8% for amino acid sequences, respectively.

## Conclusions

In our study, the association of asthma exacerbations with HRV infection appeared to be largely driven by the novel genogroup C, which was found exclusively in case-patients, and species A. The association was statistically significant for species A (detected in 15 [23%] of 65 case-patients vs. 3 [4%] of 77 controls; OR 7.4; 95% CI 1.9–43.1; p<0.001) and for genogroup C (detected in 8 [12%] case-patients vs. 0 controls; OR undefined; p<0.010) but not for infrequently identified species B (detected in 1 [2%] case-patient vs. 2 [3%] controls, p>0.05) or for HRVs that could not be genotyped (15 [23%] cases vs. 9 [12%] controls; p>0.05). The distribution of HRVs between case-patients and controls still differed when the analysis was limited to the HRV-positive group (p = 0.05) or to genotyped HRVs only (p<0.05). The results of the only other study that reported novel HRVs in asthma patients (2 of which, NAT001 and NAT045, were related to genogroup C viruses in our study) are difficult to interpret because that study of adults with “cold” symptoms showed an unexpected lack of association of HRVs with asthma exacerbation (*8*).

Patients infected with genogroup C HRVs had lower forced expiratory volumes during the first second (FEV1) than did those infected with other HRVs (median 58.5% vs. 93%; p = 0.01), but the distribution of demographic and other clinical variables did not differ significantly between the 2 groups. Lower FEV1 with genogroup C infection than with other HRVs suggests a potentially greater severity of asthma exacerbation in patients infected with these HRVs. When one considers the great variation among HRV serotypes in levels of sensitivity to candidate antiviral compounds (*12*,*13*), genogroup-related differences in associated disease patterns have implications for clinical management of HRV infections in asthma patients and for development of antiviral drugs against HRVs. Preliminary data suggest that HRV-QPM and related HRV-C strains from Hong Kong share certain VP1 sequence characteristics with HRVs that are resistant to a candidate antipicornavirus drug, pleconaril (*4*,*6*,*13*). These data raise the possibility that these novel HRVs might also be resistant to this compound.

The HRV-positive specimens from which VP1 gene sequences could not be obtained derived predominantly from patients without symptoms of acute respiratory viral illness. The absence of symptoms in HRV-infected persons likely reflects subclinical, asymptomatic infection, which is common for HRVs (*14*), or HRV persistence after a recently resolved infection (*15*), both of which are likely associated with lower viral loads (as opposed to acute symptomatic infections), thus leading to lower detection rates in a VP1 assay that uses highly degenerate primers.

In conclusion, we found a striking genetic diversity of HRVs among children with asthma and confirmed the existence and wide geographic distribution (USA, Australia, Hong Kong) of HRVs distinct from both previously recognized HRV species, A and B. Our finding supports the role of the novel HRVs as human pathogens. Additional studies are needed to further explore clinical and public health implications of these findings.
